# Reducing Dental Anxiety in Children Using a Mobile Health App: Usability and User Experience Study

**DOI:** 10.2196/30443

**Published:** 2023-10-27

**Authors:** María del Carmen del Carmen, Daniel Cagigas-Muñiz, Rocío García-Robles, Andreea Madalina Oprescu

**Affiliations:** 1 Universidad de Sevilla Sevilla Spain; 2 Department of Architecture and Computer Technology Universidad de Sevilla Sevilla Spain; 3 Department of Electronic Technology Universidad de Sevilla Sevilla Spain

**Keywords:** dentistry, dental anxiety, children, mobile, app, usability, user experience, human-centered design, mobile health, mHealth, digital health, mobile application, application development

## Abstract

**Background:**

Dentistry interventions cause common anxiety and fear problems in children (6-11 years), and according to scientific evidence, this causes a decrease in their quality of life. Therapies mediated by IT-based tools have been shown to positively influence children’s mood based on distraction as well as relaxing activities, but there is no evidence of their use to reduce dental anxiety in children.

**Objective:**

The aim of this study was to answer the following research question: Does our new children-centered codesign methodology contribute to achieving a usable mobile-based product with a highly scored user experience?

**Methods:**

A mobile health app was developed to reduce dental anxiety in children using rapid application development following the usage-centered design methodology. Structured interviews were conducted to test the usability and user experience of the app prototype among 40 children (n=20, 50%, boys and n=20, 50%, girls; age 6-11 years) using a children-adapted questionnaire and the 7-point Single Ease Question rating scale. The Smiley Faces Program—Revised questionnaire was used to assess the level of dental anxiety in participants.

**Results:**

There were no significant differences between girls and boys. The task completion rate was 95% (n=19) for children aged 6-8 years (group 1) and 100% (n=20) for children aged 9-11 years (group 2). Group 1 found watching the relaxing video (task C) to be the easiest, followed by playing a video minigame (task B) and watching the narrative (task A). Group 2 found task C to be the easiest, followed by task A and then task B. The average time spent on the different types of tasks was similar in both age groups. Most of the children in both age groups were happy with the app and found it funny. All children thought that having the app in the waiting room during a dental visit would be useful.

**Conclusions:**

The findings confirmed that the app is usable and provides an excellent user experience. Our children-adapted methodology contributes to achieving usable mobile-based products for children with a highly scored user experience.

## Introduction

### Background

Dental anxiety is a well-documented problem in scientific literature, with a high incidence in the everyday clinical practice of dentistry. It can lead to the avoidance of dental care and poor oral health outcomes in both adults and children. Managing dental anxiety from an early age can be helpful to decrease its effects and improve not only present but also future dentist experiences. However, there are only a few studies on how IT, mobile-based specifically, can help mitigate or reduce this problem in children. We have been studying this problem from the user-centered design (UCD) perspective in the App for Reducing Children’s Anxiety in Dentistry Environment (ARCADE) project. We proposed our own children-adapted codesign methodology based on an experimental study with 163 children (Romero-Ternero et al, unpublished data, August 2021). Our study concluded satisfactorily, since we checked that our methodology facilitates the UCD process and is flexible enough to be transferred to other codesign contexts with children in the health domain. Subsequently, the next steps are (1) to verify that the resulting product is usable and (2) to analyze the user experience on the mobile-based product obtained from applying the methodology. This work describes in detail the usability and user experience assessment that was carried out on a total of 40 children (n=20, 50%, girls and n=20, 50%, boys) aged 6-11 years. We analyzed the results and concluded that our methodology contributes to achieving highly scored usable mobile-based products.

### Dental Anxiety and Mobile Health

Dental anxiety is defined as a heightened fear in which the threat is unclear, ambiguous, or not immediately present [1,2]. It has a significantly negative impact on children’s oral health–related quality of life [3-5]. Children with dental anxiety are likely to avoid dental health, resulting in an increase in untreated deteriorations and cavities [6-10]. Not only physical problems but also emotional, cognitive, and behavior disorders may be caused by childhood dental anxiety [11]. These include increased pain perception, changes in mood, barriers to social relationships, sleep disorders, and low self-esteem [12,13]. This impact could be extended throughout life since untreated childhood dental anxiety is likely to continue into adolescence and adulthood.

Dental anxiety also impacts health professionals who treat children with dental anxiety as these children often have a less cooperative attitude when visiting the dentist [14,15]. It can also result in a demand for longer treatments and more resources, increasing health professionals’ stress and leading to an unpleasant experience for both patient and dentist [16,17].

The prevalence of dental anxiety is estimated to occur in 6%-20% of children and young people aged 4-18 years based on published studies [7,14,18]. Some studies [8,19] have reported even higher rates, finding that 38% and 74.1% of children have moderate or severe dental anxiety, respectively.

Numerous approaches to manage pediatric dental anxiety have been proposed, including pharmacological and nonpharmacological strategies. Among nonpharmacological strategies, behavioral techniques are commonly used because they are safe, inexpensive, and effective [9,20,21]. Distraction methods have been widely used due to their effectiveness [22]. Pretreatment education, including tell–show–do techniques, is also commonly used to reduce children’s anxiety [20,23]. Some of these techniques have been implemented using technology-based solutions, making them more attractive to children. Although these technology-based interventions are promising, there is still a lack of scientific evidence regarding their effectiveness [24]; therefore, further clinical trials must be conducted.

As an example of technology-based solutions, virtual reality distraction systems have been shown to be a successful behavior modification method in children undergoing dental treatments [25-29]. Among the diversity of information and communication technologies, mobile technology, especially using smartphone and tablet devices, is shown as a promising alternative to manage childhood dental anxiety. Mobile devices have been used to manage children’s conditions in several health domains, proving to be a viable and effective alternative [30]. Currently, children interact with mobile devices mainly for fun, associating a positive feeling/experience with their use.

Mobile devices’ characteristics and features, such as ubiquity, reduced size and weight, ease of use, reduced cost, touchscreens, integrated speakers, and input for headphones, allow the implementation of dental anxiety behavioral techniques, enabling interventions in different contexts: during dental visits, in the waiting room, during the pretreatment stage, etc. Some studies published in scientific literature have focused on mobile oral health in children, such as Campos et al [31], who designed and evaluated an educational oral health mobile app for preschoolers. However, few studies have focused on childhood dental anxiety management through mobile technology. Meshki et al [32] conducted a study to assess the effect that playing a dental simulation game would have on pain and anxiety. Children undergoing their first dental treatment session were instructed to play the game prior to the operation. The authors used a commercially available Android simulation game, called Crazy Dentist – Fun Games 1.0, twice a day for 2 weeks before the scheduled visit. They found a significant reduction in the children’s heart rate, which might result in decreased anxiety felt during anesthetic injections and drilling. The dental team must also provide information to children explaining what is going to happen during the visit in an age-appropriate way [27]. This information is considered an important part of pretreatment education and thus may be included as content in the mobile solution. Therefore, all materials designed to be used to support the management of children with dental anxiety must be adapted to their needs, skills, and preferences.

### ARCADE Project

**Figure 1:**
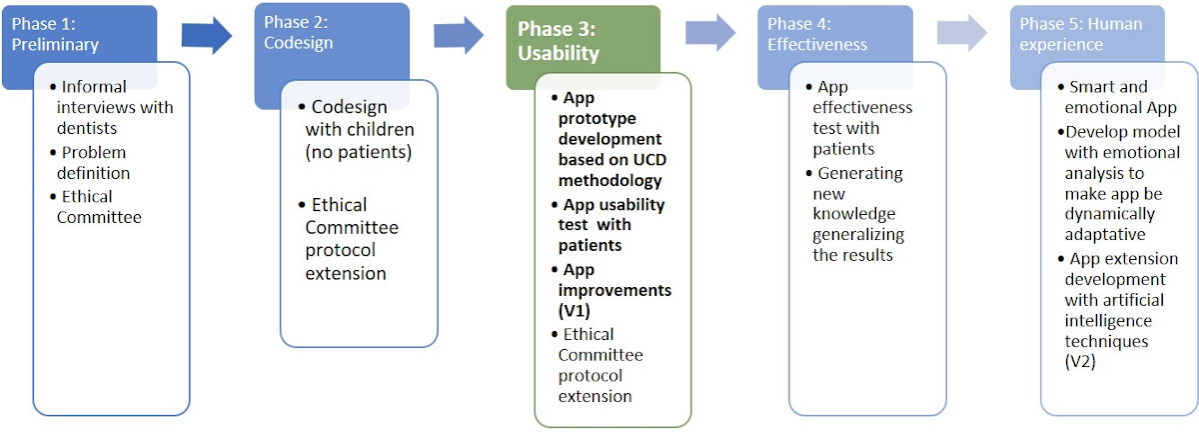
ARCADE project phases (scheme). This study deals with phase 3: app development and usability and user experience testing (green). ARCADE: App for Reducing Children’s Anxiety in Dentistry Environment; UCD: user-centered design.

The ARCADE project aims to study the feasibility of the participatory design and development of a technological solution to reduce dental anxiety problems in children aged 6-11 years. This project has a total of 5 phases, as Figure 1 shows. The first phase was described by Romero-Ternero et al [33] by defining the problem. The second phase (Romero-Ternero et al, unpublished data, August 2021) defined a new codesign method based on Druin and coworkers [34-38] and Fails and coworkers [39-41].

According to Hourcade [42], a sensible goal when designing technologies for children is to make them “child friendly.” It is important to remember the needs of children who are atypical in their sensory, physical, or cognitive abilities. In this sense, content design is a relevant stage in the development process of mobile solutions for children. Attractive and appropriate content raises user adoption and engagement rates in an anxiety management strategy. A UCD approach can be combined with age-appropriate participatory techniques to increase the levels of acceptance and satisfaction among target users [43-46]. Therefore, involving children in the design of technological solutions to support dental anxiety management in children is a crucial factor, especially in the content design stage.

Technology-based strategies for childhood dental anxiety management found in scientific literature often use age-adapted content but are not designed specifically for children with dental anxiety. Therefore, the second phase focused on creating app content by applying our codesign methodology with 163 participants between ages of 6 and 12 years (n=24, 14.7%, children from the technological summer school plus n=139, 85.3%, children from 3 different levels of a primary school). First, we concluded that the proposed methodology could obtain the adequate functionalities for an app that can be used to reduce dental anxiety in children. Second, it allowed rapid and personalized prototyping when the mobile app design was driven by the content.

This paper deals with phase 3, which seeks to study the usability and user experience of the app prototype in a population of 40 children (n=20, 50%, boys and n=20, 50%, girls) aged 6-11 years. Therefore, our goal now in phase 3 is to answer the following research question: Does our new children-centered codesign methodology contribute to achieving a usable mobile-based product with a highly scored user experience?

### Human-Centered Design: Theoretical Framework

According to International Organization for Standardization (ISO) 9241 standards [47], “Human-centered design is an approach to interactive systems development that aims to make systems usable and useful by focusing on the users, their needs and requirements, and by applying human factors/ergonomics, and usability knowledge and techniques. This approach enhances effectiveness and efficiency, improves human well-being, user satisfaction, accessibility and sustainability; and counteracts possible adverse effects of use on human health, safety and performance.”

We adopted the terms “usability” and “user experience,” as defined in the ISO 9241 standards: “Usability is the extent to which a product can be used by specified users to achieve specified goals with effectiveness, efficiency, and satisfaction in a specified context of use” and “User experience is the set of user’s perceptions and responses that result from the use and/or anticipated use of a system, product or service,” including the users’ emotions, beliefs, preferences, perceptions, comfort, behaviors, and accomplishments that occur before, during, and after use [47].

Looking to achieve these goals, the design team should adopt a human-centered design method from the beginning. As stated by Gould and Lewis [48,49], the design team needs to (1) have an early and continuous focus on users, (2) perform empirical redesign, (3) perform iterative design, and (4) perform integrated design.

There are many possible professional methods for undertaking such a design process. However, as mentioned before, the ARCADE project focuses on children. In this context, “usage-centered design” by Constantine and Lockwood [50] was selected due to the following implicit features: (1) It is a minimalist method, which supports rapid user prototyping involving users, and (2) due to its simplicity, it is a suitable method to be used with children, the target group of the ARCADE project. The usage-centered design, as used in the ARCADE project, is further explained in the “Design Methodology” section.

In relation to usability testing, a variety of techniques enable developers to identify the problems and shortcomings in software. Inspections and review offer quick, efficient, and easy methods to find usability problems and help identify potential solutions. Usability inspections and reviews complement good user interface engineering by finding problems and limitations.

In the framework of this project, 1 type of “structured inspections” was applied to identify usability defects. Specifically, the “collaborative usability inspection” method was selected due to several reasons. First, it is versatile and can be easily applied to a functional prototype. Second, usability inspection is a set of focused activities that can be introduced into almost any software development process without involving many resources. Finally, usability inspections are easily learned, and this can be useful when these inspections need to be accomplished with children as the main target user group.

Compared to most testing schemes, inspections are faster and simpler to apply. Inspections are also more efficient than other schemes because they imply organization, a systematic process, and focus.

## Methods

### Design Methodology

We used the usage-centered design methodology to design ARCADE. According to Constantine and Lockwood [50], *structured inspections* are organized activities with specific roles and responsibilities. They are carried out in orderly, step-by-step processes that ensure important activities are completed and that help keep attention from being diverted. Roles, such as software developers, end users, domain experts, and usability specialists, collaborate to perform an in-depth and efficient inspection of a finished product in *collaborative usability testing* sessions. By drawing representative inspections of the user community directly, the user-centered perspective is incorporated into the process and some of the benefits of more expensive and elaborate objective usability testing can be gained through simple inspections.

Collaborative usability testing is a quality assurance tool. The ultimate objective of the inspection is to improve product quality in terms of usability. Developers must keep in mind that the purpose of the inspection is to identify defects. “Finding defects is not a sign of failure but of success” [50-52].

In the context of ARCADE, the primary purpose was to identify usability defects and interface inconsistencies in the functional prototype. The group carrying out the collaborative usability inspection, referred to as the inspection team, comprised software development leaders and end users (children in the specified age range). The usability testing sessions were scheduled to be held in teams of 3 people, 2 (66.7%) members of the software development team and 1 (33.3%) child. A significant number of sessions with different children and rotating developers were undertaken.

More specifically, the inspection roles involved (1) the *lead reviewer*, the researcher who led the inspection method; (2) the *inspection recorder*, the researcher responsible for keeping a complete record of all defects identified, noting their location in the system, and summarizing the problem; and (3) the *user*.

The inspection process consisted of 4 stages: (1) preparation, (2) interactive inspection, (3) static inspection, and (4) finalization and follow-up.

Considering that we interviewed 40 children, the amount of time we spent during each phase was as follows: preparation: 3 weeks; interactive inspection: 7 weeks (3 weeks + COVID-19 pandemic interruption + 4 weeks); static inspection: 2 weeks; and finalization and follow-up: 4 weeks.

In the *preparation phase,* the tasks to be undertaken by the user with the functional prototype were selected and accurately described. These tasks were prepared in advance as usage scenarios that included typical characteristics and critical interactions in normal system usage.

In the *interactive inspection phase*, the system was used to carry out the aforementioned representative tasks. After each task scenario was completed, the lead reviewer asked for comments on what features of the current design seemed to work effectively and should be preserved.

In the *static inspection phase*, the inspection team reviewed all interaction scenarios and the usability defects detected were further documented. In the *finalization and follow-up phase,* the usability defects were discussed and some improvements were identified and communicated to the developer team to enhance the app functional prototype. In addition to the inspection process (collaborative usability testing), a user experience questionnaire was used to reach some conclusions about the engagement and overall impression of the app by the end users. Usage-centered design is a model-driven process using 3 primary abstract models: a user role model, a task model, and a content model [50]. The user role model captures and organizes selected aspects of particular users and the system being designed. The task model represents, in the form of essential use cases, those things that users in user roles are interested in accomplishing with the system. Finally, the content model represents the content and organization of the user interface, in addition to its appearance and behavior.

**Figure 2:**
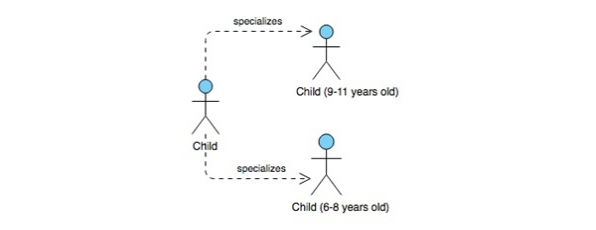
ARCADE project usage-centered design user roles map. ARCADE: App for Reducing Children’s Anxiety in Dentistry Environment.

**Figure 3:**
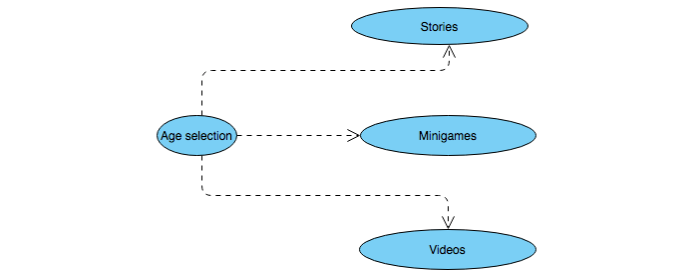
ARCADE project usage-centered design use case map. ARCADE: App for Reducing Children’s Anxiety in Dentistry Environment.

Each of these 3 models consists of 2 parts: a collection of descriptions and a map of the relationships among those descriptions [53]. The unified modeling language maps are the user role map, the use case map, and the navigation map. A few samples of the maps applied to the app design are shown in Figures 2 and 3.

Tasks carried out in usability tests and the app improvements detected are detailed in the “Results” and “Discussion” sections, respectively.

### Development Methodology

The app was developed using the rapid application development (RAD) methodology, originally described by Martin [54]. This methodology prioritizes rapid prototyping and user participation. There are 4 main phases in RAD: (1) requirements planning (project goals and scope, expectations, timelines), (2) user design (workshops and meetings with users to model the system), (3) construction (building the designed prototype into functional software, incorporating user feedback via iterations), and (4) cutover (finalized prototype). The RAD methodology has been previously used in conjunction with the UCD principle, including it as an additional phase of the methodology [55].

This methodology requires clear goals and requirements, a modular design, experienced developers, and user involvement. Therefore, this methodology fit our software development process for several reasons: (1) The requirements of the prototype were clearly defined at the beginning of the development process, (2) the app presents a modular structure as we defined each resource as a separate module, (3) we had an experienced developer in our team, and (4) there was high involvement of the user throughout the UCD process. As a result, we obtained a rapid functional prototype to test with the children.

### Usability and User Experience Test Methodology

Once ARCADE was available, its usability and user experience were assessed using structured interviews of 40 children aged 6-11 years during the *interactive inspection* (see the “Design Methodology” section). Usability and user experience test descriptions and the results obtained are described later.

#### Data Collection and Sample

In total, 40 children were recruited from public primary schools in Seville (Spain). They were divided into 2 main groups: 20 (50%) boys and 20 (50%) girls. The children were also divided into 2 groups according to age: 20 (50%) children aged 6-8 years (n=10, 50%, boys and n=10, 50%, girls) and 20 (50%) children aged 9-11 years (n=10, 50%, boys and n=10, 50%, girls). None of the children had previously used the app. According to Yáñez Gómez [56], an average of 10 users’ evaluation is enough for a usability test. In this study, differences between children’s ages and genders were analyzed.

The evaluations took place in different settings: 2 faculties of the University of Seville and primary schools.

#### Selection Criteria

The inclusion criteria for the evaluated population were:

- Age: The children had to match 1 of the 2 age ranges.
- No reading disabilities.
- Previously use of mobile devices: tablets or smartphones.
- Not used ARCADE before.

#### Material

The materials used in usability and user experience tests were:

- A tablet with the following specifications: 10 inches, HD IPS, 800×1280 pixels; Android 10; device: DUODUOGO, Quad -Core 1.3GHz, 4GB RAM, 64 GB ROM
- ARCADE [57]
- A usability and user experience questionnaire to fill in by researchers during interviews [57]
- A chronometer

#### Intervention

To verify the correct usability of ARCADE, the ISO 9241-11 standard [47] was followed. This standard considers the ergonomics and usability of terminals with display screens. The ISO 9241-11 framework makes it possible to visualize all factors that can affect the usability of a system in use, from a real perspective, which is fundamental in determining real user needs [58].

Specifically, the ISO 9241-11 standard proposes the evaluation of usability through 3 factors: (1) effectiveness (accuracy and completeness with which users achieve specific goals), (2) efficiency (resources expended in relation to the accuracy and completeness with which users achieve goals), and (3) satisfaction (comfort and acceptability of use).

According to Hourcade [42], usability goals that are regularly used in usability studies with adults, such as efficiency and effectiveness, also apply to children’s low-level interactions with technologies. Usability professionals typically measure usability goals through usability testing, which involves children completing specific tasks while their behavior is recorded and measured.

The usability of ARCADE was evaluated using structured interviews, according to the ISO 9241-11 standard, by means of 3 tasks given to children:

- Task A: Select and read a narrative. The narratives have a different number of possible endings. Some narratives have only 1 ending, while others have 2, 3, or 5 possible endings. When opening a narrative, a pop-up with selectable options according to the number of possible endings is displayed in ascending order. The lead reviewer indicated to the children which specific narrative to select depending on the age group to which they belonged. Children aged 6-8 years (group 1) were indicated to select a narrative with only 1 possible ending. Children aged 9-11 years (group 2) were indicated to select a narrative with 2 possible endings; they were asked to select the second ending. Selected narratives for both groups had the same number of screens.
- Task B: Select and finish a card-matching memory video minigame.
- Task C: Select a multimedia file (a relaxing video and a story told by a child).

For each task, the following were evaluated:

- Effectiveness: We checked whether each task could be completed (ie, select and finish the correct narrative game and video).
- Efficiency: We measured the time spent on each task.
- Satisfaction: We initially considered the System Usability Scale (SUS) [59] to evaluate the satisfaction part of usability. This questionnaire comprises 10 questions with 5 possible answers, depending on the degree of conformity with the answer. The SUS has become an industry standard. However, the SUS is not designed for use with children. There is no clear guideline or consensus in scientific literature about its suitability for use in usability tests with children (ie, there is no consensus on the minimum age at which the SUS can be used). There are some examples of the application of the SUS in children, but generally, the population is small [60,61] or the children generally have an average age of about 13 years [62,63]. The SUS was initially tested in the ARCADE project with 3 children 7 and 8 years of age. It was clearly concluded because of contradictions in the answers provided that the children did not understand some questions well. For example, SUS questions 2 and 3 had completely opposite evaluations.

I found the system unnecessarily complex.

I thought the system was easy to use.

**Figure 4:**
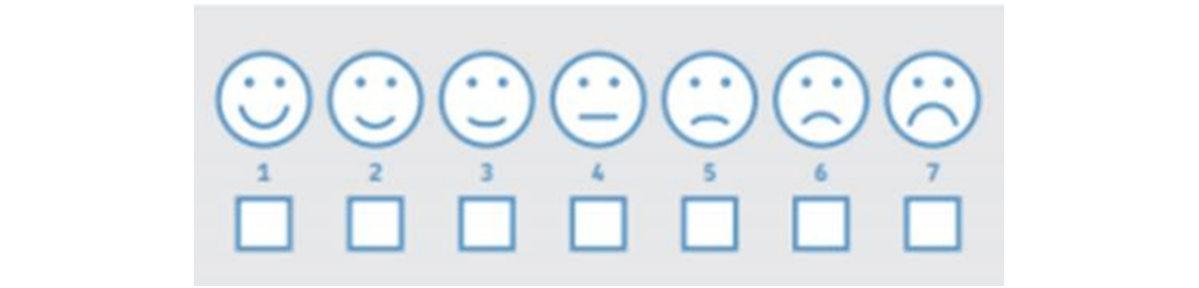
SEQ pictogram. SEQ: Single Ease Question.

All these conclusions forced us to redesign the satisfaction usability evaluation part. The satisfaction evaluation was carried out at the task level using a final satisfaction test of the whole app. In both cases, standardized satisfaction questionnaires were used. For each task, the Single Ease Question (SEQ) [64] was used. The SEQ is a 7-point rating scale used to assess how difficult users find a task. In this case, a face scale pictogram was provided to children to facilitate their answering (see Figure 4).

The global app satisfaction test was evaluated using the 4 questions of the satisfaction part [65], which were adapted from evidence-based considerations in evaluating the usability of mobile health (mHealth) tools [66] and also applied to children:

- Are you happy with the app?
- Would you tell a friend about the app?
- Is the app funny?
- Has the app worked as you expected?

Finally, at the end of the test session, we asked each child whether they thought that having the app in the waiting room during a dental visit would be useful. This was an informal question to better understand their general opinion about the app and its future acceptance.

### Dental Anxiety Test Methodology

Children participating in this study were not necessarily those with dental anxiety, so we did not measure the effectiveness of this app in reducing dental anxiety. We measured its usability and user experience with children in the same groups as the target children. However, to check whether this population was representative of a population with dental anxiety, we assessed the general level of dental anxiety of each participant using the 5-item Smiley Faces Program—Revised (SFP‐R) questionnaire [67]:

- If you had to go to the dentist tomorrow, how would you feel?
- If you were sitting in the dentist’s waiting room, how would you feel?
- If you were about to get a hole in your tooth, how would you feel?
- If you were about to get an injection of anesthesia in the gum, how would you feel?
- If the dentist were about to extract a tooth, how would you feel?

Each question has 7 options numbered from 1 (maximum level of agreement or satisfaction) to 7 (maximum level of dissatisfaction or disagreement). Scores range from 5 (no anxiety at all) to 35 (maximum anxiety) points. Each score has a pictogram in the form of a smiley or a sad face that is associated with each of the 7 possible answers to each question (see Figure 4). Pointing out pictograms allows children to express their answers more naturally. The minimum possible score is 5 points, and the maximum is 35. A high score denotes evidence of dental anxiety. However, there is no formal limit at which it is determined that the child has dental anxiety.

### Ethical Considerations

Prescriptively, an information sheet and an informed consent form were provided to the legal guardians of the participants in this study. The information and personal data of the participants was kept completely confidential with all the rigor of the law. The activities, signed consent forms, and questionnaires were approved by the Andalusian Ethical Committee.

## Results

### Prototype

ARCADE is an Android app written in Java via Android Studio (Google, JetBrains). The app opens with a start screen, on which the user is presented with different tasks: narratives, video minigames, and relaxing videos and audio stories. Once the user selects the category of choice by tapping the panel, a list of all available items within the category is displayed. The navigation through all the items is achieved by scrolling, and a resource can be selected by tapping its panel. Each view has a return button in the upper left corner.

Navigation through the narratives is achieved by swiping left (go to the next page) or right (return to the previous page). The relaxing videos with the storytelling start automatically when selected from the list. A progress bar is placed at the bottom of the videos.

The main ARCADE screens and author translation of the text on the screens can be found in Multimedia Appendix 1.

### Outcomes

The task completion rate was 95% (n=19) for children aged 6-8 years (group 1) and 100% (n=20) for children aged 9-11 years (group 2); 1 (5%) girl did not complete the narrative task (she selected the wrong one) because of her reduced reading skills.

Efficiency was measured through the task completion time. Table 1 shows a summary of the time spent on different tasks by age range.

**Table 1:** Time in seconds spent to complete the 3 tasks: select and read a narrative (task A), select and finish a card-matching memory video minigame (task B), and select and listen to a relaxing video (task C).

Task	Group 1 (6-8 years old), mean (SD)	Group 2 (9-11 years old), mean (SD)
A	165 (72)	173 (70)
B	113 (42)	101 (38)
C	60 (25)	75 (37)

#### Usability and User Experience Data Analysis

**Figure 5:**
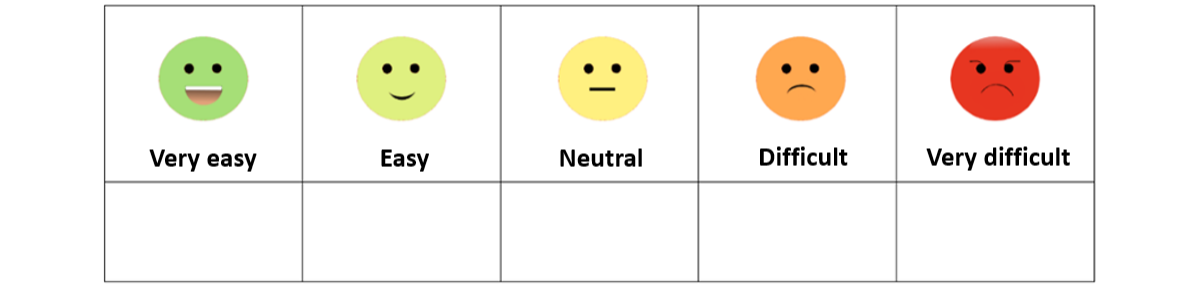
The 1-5 face scale ranging from 1 (very easy) to 5 (very difficult).

We analyzed the results of the usability and user experience tests by dividing the children by age into 2 groups: 6-8 years old (group 1) and 9-11 years old (group 2). Children were asked to rate how easy they found each task, on a 1-5 face scale ranging from 1 (very easy) to 5 (very difficult); see Figure 5.

Children from group 1 spent an average of 165 (SD 72) seconds to complete the narrative task (task A), 113 (SD 42) seconds on the video minigame task (task B), and 60 (SD 25) seconds on the relaxing video task (task C). Children from group 2 spent an average of 173 (SD 70) seconds to complete task A, 101 (SD 38) seconds to complete task B, and 75 (SD 37) seconds to finish task C. These results can be seen in Table 1.

The average time spent on the different types of tasks was similar in both groups. On average, all children spent the maximum time on task A, followed by task B. Lastly, the shortest task for all children was watching the relaxing video (task C).

**Figure 6:**
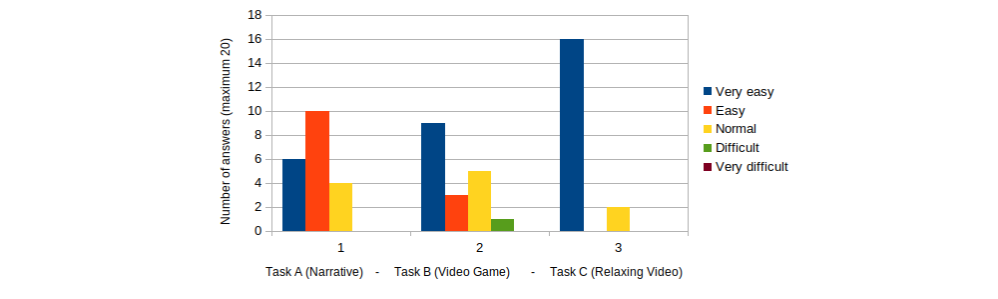
SEQ results for children aged 6-8 years (group 1). SEQ: Single Ease Question.

**Figure 7:**
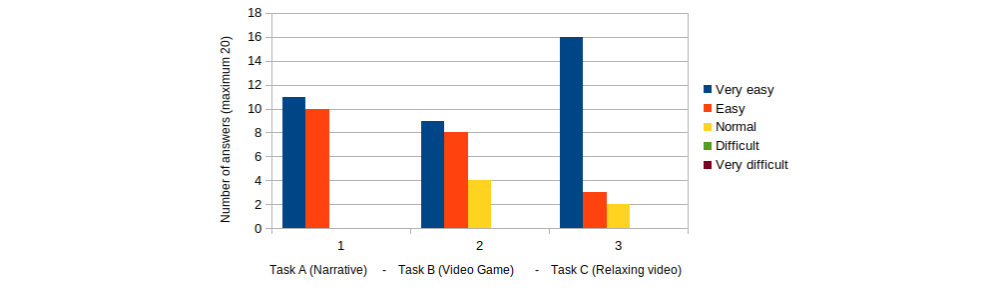
SEQ results for children aged 9-11 years (group 2). SEQ: Single Ease Question.

**Figure 8:**
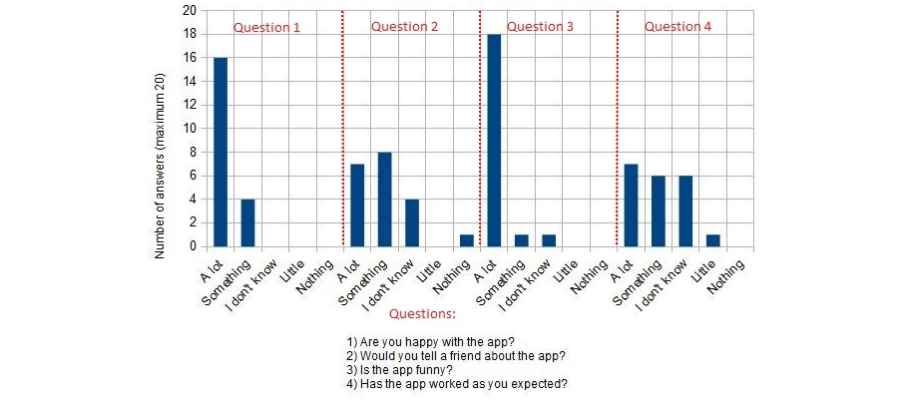
ARCADE general satisfaction results for children aged 6-8 years (group 1). ARCADE: App for Reducing Children’s Anxiety in Dentistry Environment.

**Figure 9:**
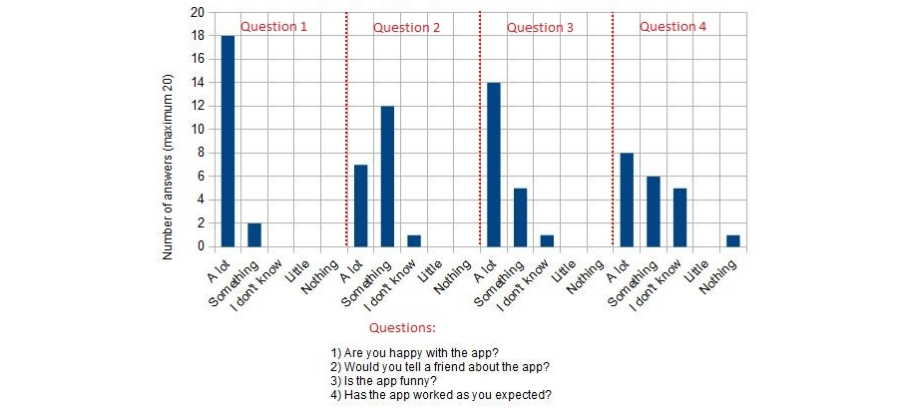
ARCADE general satisfaction results for children aged 9-11 years (group 2). ARCADE: App for Reducing Children’s Anxiety in Dentistry Environment.

Results of the SEQ satisfaction test are shown in Figure 6 (group 1) and Figure 7 (group 2). Results of the general satisfaction questionnaire are shown in Figure 8 (group 1) and Figure 9 (group 2).

When children in group 1 were asked to rate the different tasks, 6 (30%) children rated task A as very easy, 10 (50%) as easy, and 4 (20%) as normal. The minigame task B) was evaluated as very easy by 11 (55%) children, easy by 3 (15%) children, and normal by 5 (25%) children; 1 (5%) child found the task to be difficult. In addition, task C was found to be easy by 18 (90%) children and normal by 2 (10%) children. In this age group, none of the children found any task to be very difficult. These results are shown in Figure 6.

From these results, we observed that children in group 1 found task C to be the easiest task, followed by task B. Watching the narrative (task A) was mostly found to be easy. In comparison, of the 3 tasks, task A was found to be the least easy.

Children in group 2 were also asked to rate how easy they thought the different tasks were. In this case, 10 (50%) children rated task A as very easy and 10 (50%) rated it as easy. Task B was found to be very easy by 9 (45%) children, easy by 7 (35%) children, and normal by 4 (20%) children. Task C was rated as very easy by 15 (75%) children, easy by 3 (15%) children, and normal by 2 (10%) children. In this age group, none of the children found any task to be difficult or very difficult. These results are shown in Figure 7.

From these results, we perceived that most children in group 2 found task C to be the easiest task. Tasks A and B were also deemed easy.

All children found task C to be the easiest. Task A was rated easier by the children in group 2 than the children in group 1.

As mentioned in the “Intervention” section, the children were asked 4 questions as part of the general satisfaction questionnaire [65]: (1) Are you happy with the app? (2) Would you tell a friend about the app? (3) Is the app funny? (4) Has the app worked as you expected?

When asked whether they were happy with the app, most of the children (n=16, 80%) in group 1 answered “a lot” and 4 (20%) answered “something” (see Figure 8). The children had mixed opinions when asked whether they would tell a friend about the app: 7 (35%) answered “a lot,” 8 (40%) answered “something,” and 4 (20%) did not know whether they would. When asked whether they found the app to be funny, most of them (n=18, 90%) replied affirmatively, 1 (5%) child replied “something,” and 1 (5%) child said they did not know. There were also some mixed results regarding the last question, whether the app worked as they expected: 7 (35%) children answered “a lot,” 6 (30%) answered “something,” 6 (30%) did not know, and 1 (5%) answered “just a little.” When the children who answered “I don’t know” (n=6, 30%) or “just a little” (n=1, 5%) were asked why, they explained the app exceeded their expectations.

The first question got similar results from the children in group 2 (see Figure 9). Most children (n=18, 90%) answered “a lot”, and 2 (10%) answered “something.” When asked whether they would tell a friend about the app, 7 (35%) children answered “a lot” and most of them (n=12, 60%) said “something”; 1 (5%) child did not know whether they would. More than two-thirds of the children (n=14, 70%) responded “a lot” when asked whether they found the app to be funny, 5 (25%) answered “something,” and 1 (5%) child did not know. Like the children in group 1, children in this group had mixed feelings about the app working as they expected. Nearly half of the children (n=8, 40%) said it worked a lot as they expected, 6 (30%) children responded that it worked something like how they expected, and 5 (25%) said they did not know. Only 1 (5%) child responded that the app worked nothing like expected.

After the test session, when asked whether they thought that having the app in the waiting room during a dental visit would be useful, all children answered affirmatively.

Expert observations during tests and some questions asking children to better explain their answers revealed some problems. First, some children did not know how to navigate through the narratives. They had to find out how to continue reading and reach the end. Second, most children in both age groups expected the videos playing while listening to the relaxing story to finish or stop when the story was over.

#### Dental Anxiety Data Analysis

**Figure 10:**
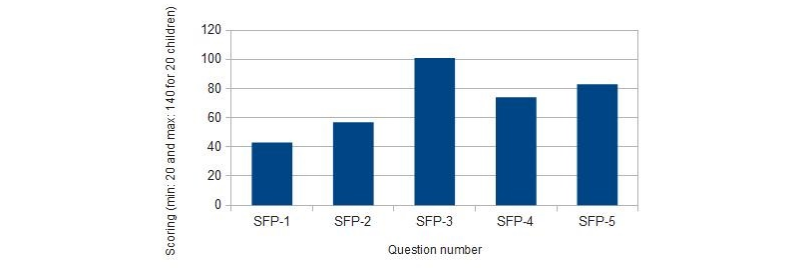
Smiley Faces Program—Revised (SFP-R) questionnaire results for children aged 6-8 years (group 1).

**Figure 11:**
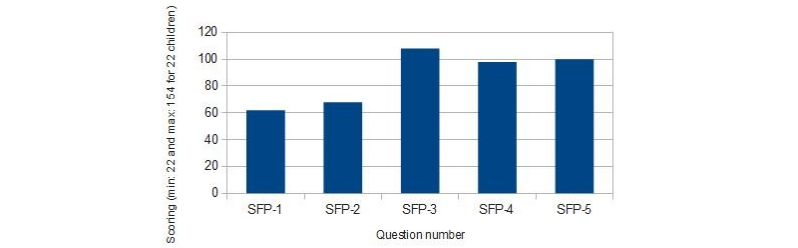
Smiley Faces Program—Revised (SFP-R) questionnaire results for children aged 9-11 years (group 2).

Regarding the assessment of dental anxiety in participants, Figure 10 (group 1) and Figure 11 (group 2) show the results obtained. To analyze SFP-R results, the scores of each child were divided into quartiles considering gender and age.

**Figure 12:**
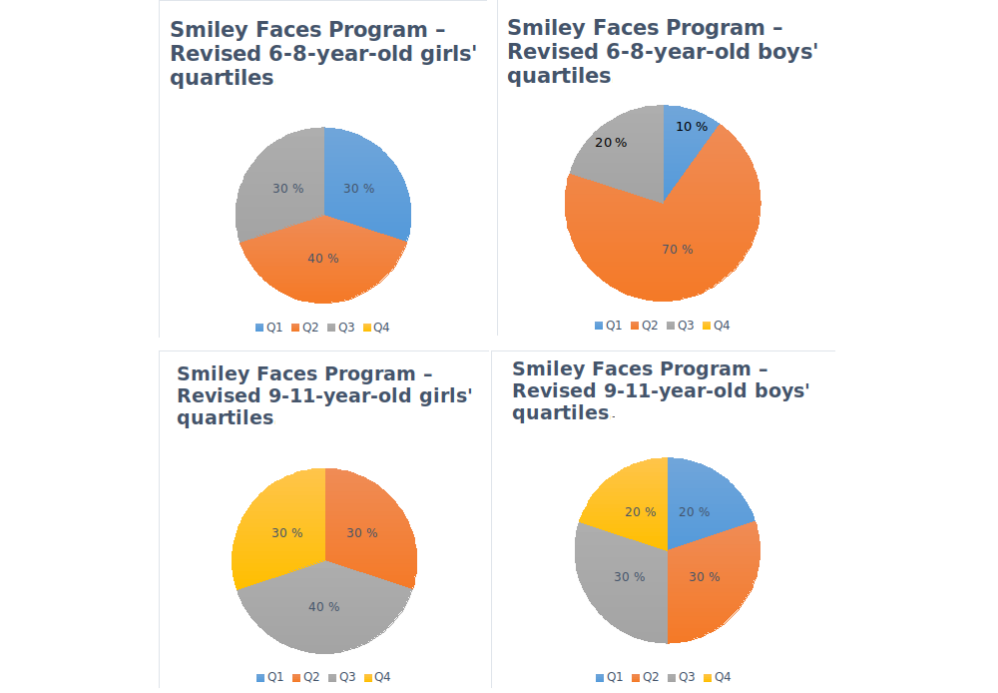
Smiley Faces Program—Revised (SFP-R) questionnaire quartile results for girls (left) and boys (right) between 6-8 (top) and 9-11 (bottom) years.

Figure 12 shows a summary by gender (girls left and boys right) and age (group 1 top and group 2 bottom), where Q1 represents the lowest dental anxiety and Q4 the highest. In the case of girls aged 6-8 years, 3 (30%) were in Q1, 3 (30%) were in Q2, and 4 (40%) were in Q3. In the case of girls aged 9-11 years, 3 (30%) were in Q2, 4 (40%) were in Q3, and 3 (30%) were in Q4. In the case of boys aged 6-8 years, 1 (10%) was in Q1, 7 (70%) were in Q2, and 2 (20%) were in Q3. Finally, the case of boys aged 9-11 years, 2 (20%) were in Q1, 3 (30%) were in Q2, 3 (30%) were in Q3, and 2 (20%) were in Q4.

## Discussion

### Principal Findings

The results of the usability test showed high scores for the 3 evaluated aspects: (1) regarding effectiveness, the app is easy and effective as all except 1 child completed all 3 tasks; (2) in terms of efficiency, children spent a short amount of time to achieve each task; and (3) children exhibited high satisfaction after using the app.

Despite these positive results, during the tests, we identified 2 usability problems in the app: the first problem with the narrative and the second problem with the relaxing video with audio stories. The first problem was that most children did not figure out how to navigate through the narratives. The navigation was originally implemented as simulating going through a physical book, sliding left and right to change the page. It took them several seconds until they realized that by sliding their finger to the right, they could turn the page of the narrative. One possibility to improve this usability problem is to add 2 right-left directional arrow buttons below the narrative images. With the arrow buttons, the children would know how to navigate back and forth through the different narrative screens. The second usability problem was that most children found it confusing that the video kept playing after the story was over. They waited for a while until they realized the task was over. One way to improve this usability problem is to stop the video reproduction and add progress time stamps to the video so that it is visible to the user when it is over.

Gender differences were not found. However, in the case of group 1 (6-8 years), the narrative and video minigame tasks were ranked as more difficult in the SEQ. In this case, lower reading and cognitive skills were noted during the tests.

Although the main aim of this study was to carry out usability and user experience tests of ARCADE with children, we were interested in their thoughts on the usefulness of having the app in the dentist’s waiting room. Asking this question was relevant since the app’s acceptance is a key factor for future studies on the effective reduction in dental anxiety during dental visits. All the participants said they would enjoy having the app in the waiting room.

Regarding the results of the SFP-R, girls showed more evidence of suffering some grade of dental anxiety than boys. This could be explained from a cultural perspective (*boys are brave and do not cry, nor are they afraid*). Children in the 9-11-year age range also had in general more evidence of dental anxiety than the younger children. This could be explained by the fact that older children have had in general more dental treatments or visits to the dentist.

### Limitations

A previous evaluation of dental anxiety was not performed as a selection criterion for the children participating in this study. However, we assessed the general level of dental anxiety of the participating children using the SFP‐R questionnaire. The COVID-19 pandemic was an important inhibitor in finding participants for tests. This fact delayed the usability tests for many months.

### Conclusion

This study examined the usability and user experience of a mobile-based app prototype designed for reducing children’s dental anxiety. Overall, all the children who underwent the usability and user experience tests found the app prototype to be easy to use and understand and felt happy when using the app.

The whole process for testing usability and user experience with children (6-11 years) has been described in detail. The results show that using attractive and children-adapted content can promote the adoption of the tool by children. The results are evidence that our children-adapted codesign methodology contributes to achieving a usable mobile-based product with a highly scored user experience.

The fact that the prototype app was found to be easy to use and enjoyable by the children is a key factor in its future acceptance. Additionally, several usability improvements were identified in this study following the usage-centered design methodology.

Implementing these types of human-centered design methodologies from the beginning helps achieve mHealth solutions for children with high rates of acceptance. We understand that the results of this study are positive and will enable the ARCADE project to move forward to its next phase.

Based on the positive results of this study, the following phases will consist of testing the prototype effectiveness in reducing dental anxiety in patients (phase 4 in Figure 1). It is planned that the last phase will include artificial intelligence and emotional computing to provide emotional support to patients (phase 5 in Figure 1).

## Appendix

Multimedia Appendix 1 ARCADE app screens. From top left to bottom right: introduction screen, narratives, minigames, and relaxing videos and audio stories. Authors translated the text on the screens.

## Abbreviations

ARCADE: App for Reducing Children’s Anxiety in Dentistry Environment
ISO: International Organization for Standardization
mHealth: mobile health
RAD: rapid application development
SEQ: Single Ease Question
SFP-R: Smiley Faces Program—Revised
UCD: user-centered design
