# Supplementary material for: Reducing Dental Anxiety in Children Using a Mobile Health App: Usability and User Experience Study
Source: JMIR Form Res. 2023 Oct 27;7:e30443. doi: 10.2196/30443 (PMC10638634; doi:10.2196/30443)
Supplement: Multimedia Appendix 1 [file formative_v7i1e30443_app1.docx]

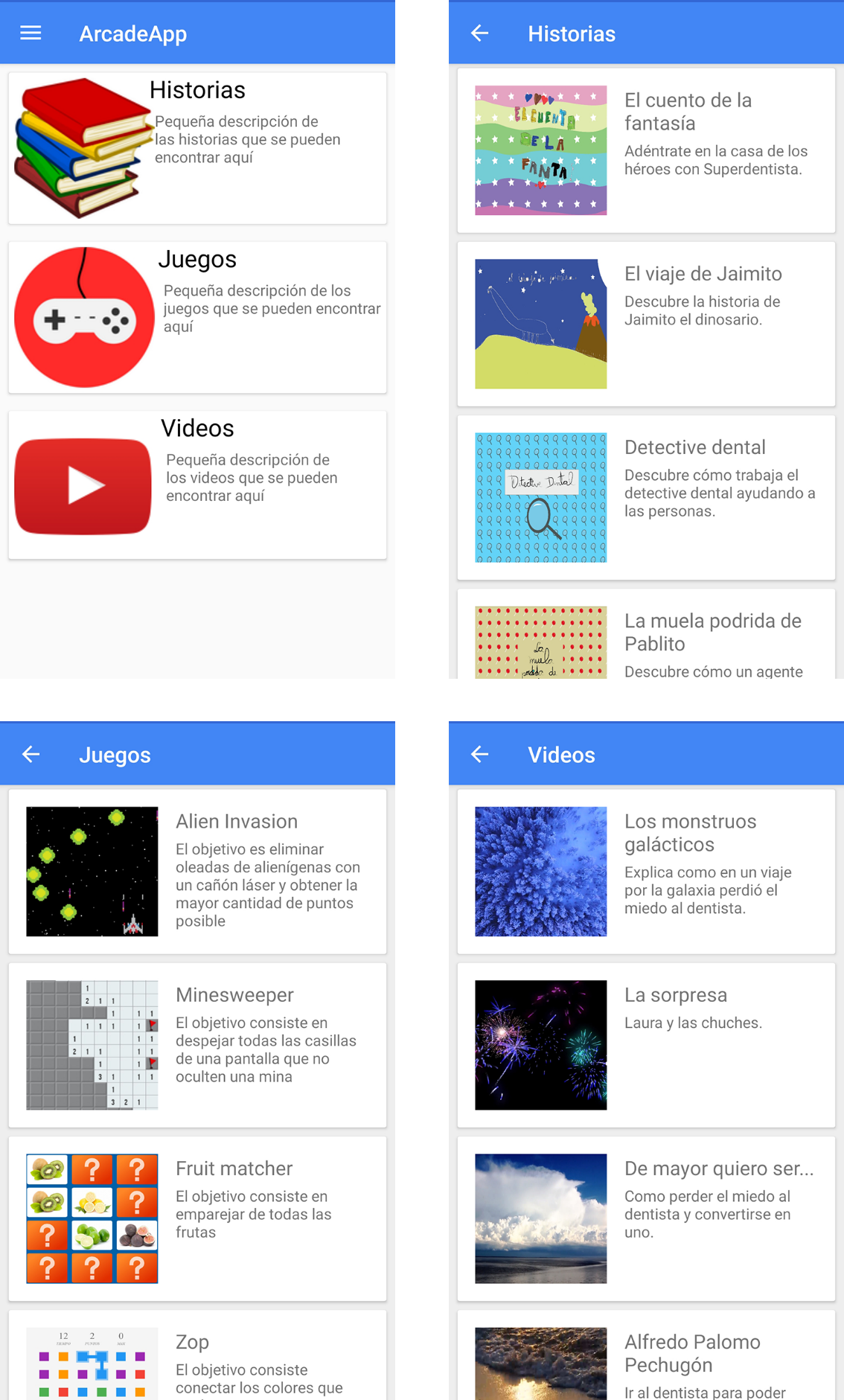


ARCADE App screens. From top left to down right: introduction screen; narratives; minigames; relaxing videos and audio-stories.

**Author translated text on screens:**

**ARCADE App.** Historias. Pequeña descripción de las historias que se pueden encontrar aquí. [ARCADE App. Stories. Short description of the stories that can be found here.]. Juegos. Pequeña descripción de los juegos que se pueden encontrar aquí. [Games. Short description of the games that can be found here.]. Videos. Pequeña descripción de los videos que se pueden encontrar aquí. [Videos. Short description of the videos that can be found here.]

**Historias.** El cuento de la fantasía. Adentrate en la casa de los heroes con superdentista. [Stories. The fantasy tale. Enter the house of heroes with a super dentist.] El viaje de Jaimito. Descubre la historia de Jaimito el dinosaurio. [James' trip. Discover the story of James the dinosaur.] Detective dental. Descubre cómo trabaja el detective dental ayudando a las personas. [Dental detective. Discover how the dental detective works helping people.]

**Juegos.** Alien invasion. El objetivo es eliminar oleadas de alienigenas con un cañón laser y obtener la mayor cantidad de puntos posibles. [Games...The objective is to eliminate waves of aliens with a laser cannon and obtain as many points as possible.] Minesweeper. El objetivo consiste en despejar todas las casillas que no oculten una mina. [The objective is to clear all the squares that do not hide a mine.] Fruit matcher. El objetivo consiste en emparejar todas las frutas. [The goal is to match all the fruits]

**Videos.** Los monstruos galacticos. Explica como en un viaje por la galaxia perdió el miedo al dentista. [Videos. The galactic monsters. Explain how on a trip through the galaxy you lost your fear of the dentist.] La sorpresa. Laura y las chuches. [The surprise. Laura and the sweets.]. De mayor quiero ser...Como perder el miedo al dentista y convertirse en uno. [When I grow up, I want to be ... How to lose my fear of the dentist and become one.]}.
